# Skimmianine Pretreatment Attenuates Cerebellar Neuroinflammation and Myelin Injury Following Experimental Cerebral Ischemia–Reperfusion

**DOI:** 10.3390/antiox15060743

**Published:** 2026-06-11

**Authors:** Fırat Aşır, Ebru Gökalp Özkorkmaz, Murat Yalçın, Fırat Şahin, Tuğcan Korak

**Affiliations:** 1 Department of Histology and Embryology, Faculty of Medicine, Dicle University, 21280 Diyarbakır, Turkey; 2 Faculty of Health Sciences, Ankara Yıldırım Beyazıt University, Çubuk, 06760 Ankara, Turkey; ebru.gokalpozkorkmaz@aybu.edu.tr; 3 Division of Psychiatry, Diyarbakır Gazi Yaşargil Training and Research Hospital, Health Sciences University, 21070 Diyarbakır, Turkey; 4 Division of Infertility, Turan Çetin In-Vitro Fertilization Center, Çukurova, 01360 Adana, Turkey; sfirat9021@gmail.com; 5 Department of Medical Biology, Medical Faculty, Kocaeli University, 41380 Kocaeli, Turkey; tugcankorak@gmail.com

**Keywords:** skimmianine, ischemia/reperfusion, cerebellum, neuroinflammation, oxidative stress, myelin injury

## Abstract

Objective: Cerebral ischemia/reperfusion (I/R) injury triggers oxidative stress, neuroinflammation, neuronal degeneration, and white matter damage not only in directly affected cerebral regions but also in remote brain areas such as the cerebellum. Skimmianine, a naturally occurring furoquinoline alkaloid, has been reported to possess antioxidant and anti-inflammatory properties. This study investigated the protective effects of skimmianine pretreatment against secondary cerebellar injury following experimental cerebral I/R. Materials and Methods: Thirty-two female Wistar rats were randomly assigned to sham, Skimmianine, I/R, and I/R + Skimmianine groups (*n* = 8/group). Cerebral I/R was induced by transient middle cerebral artery occlusion for 60 min followed by 23 h reperfusion. Skimmianine (40 mg/kg/day, intraperitoneally) was administered for 14 days before ischemia induction. Oxidative stress markers, neuroinflammatory mediators, histopathological alterations, behavioral outcomes, and ultrastructural changes were evaluated. In addition, network pharmacology and molecular docking analyses were performed to explore potential molecular mechanisms. Results: Cerebral I/R significantly decreased TAS levels compared with sham (0.89 ± 0.15 vs. 1.52 ± 0.18 mmol Trolox Eq/L) and increased TOS (15.60 ± 3.03 vs. 6.80 ± 1.41 µmol H_2_O_2_ Eq/L), OSI (17.48 ± 0.50 vs. 4.43 ± 0.47), TNF-α (68.4 ± 10.2 vs. 18.6 ± 4.4 pg/mL), Iba1 (41.3 ± 9.7 vs. 11.7 ± 1.6 pg/mL), and GFAP levels (334.5 ± 12.5 vs. 87.7 ± 9.5 ng/mL; all *p* < 0.001). I/R also impaired motor performance, as shown by increased beam crossing time (11.7 ± 2.2 vs. 4.8 ± 0.7 s) and grid foot fault rate (18.6 ± 4.0% vs. 3.4 ± 1.1%). Skimmianine pretreatment significantly improved these alterations, increasing TAS to 1.29 ± 0.20 mmol Trolox Eq/L and reducing TOS, OSI, TNF-α, Iba1, and GFAP levels to 9.20 ± 2.04, 7.07 ± 0.47, 34.9 ± 7.4, 24.2 ± 6.9, and 237.0 ± 7.9, respectively, compared with the untreated I/R group. Histopathological scores for Purkinje cell loss, edema, vascular congestion, and TNF-α expression were also significantly reduced by skimmianine. Quantitative TEM analysis showed that I/R reduced myelin thickness (0.29 ± 0.05 vs. 0.53 ± 0.07 µm), increased G-ratio values (0.75 ± 0.05 vs. 0.63 ± 0.04), and increased vacuolized fibers (24.70 ± 4.20% vs. 3.20 ± 1.10%), whereas skimmianine partially restored myelin thickness (0.42 ± 0.07 µm), reduced the G-ratio (0.68 ± 0.05), and decreased vacuolized fibers (11.20 ± 2.80%; *p* < 0.05 vs. I/R). Molecular docking demonstrated favorable binding between skimmianine and TNF-α, with a predicted binding energy of −6.953 kcal/mol. Conclusions: These findings indicate that skimmianine exerts neuroprotective effects against secondary cerebellar injury following cerebral I/R through coordinated modulation of oxidative stress, systemic neuroinflammatory responses, astroglial injury-associated pathways, and inflammation-related mechanisms.

## 1. Introduction

Cerebral ischemia–reperfusion (I/R) injury is a major pathological process underlying secondary brain damage following ischemic stroke and traumatic brain injury [1]. Although timely reperfusion is essential for restoring cerebral blood flow and limiting infarct expansion, the abrupt reintroduction of oxygen and metabolic substrates paradoxically initiates a cascade of deleterious events, including excessive reactive oxygen species (ROS) production, mitochondrial dysfunction, disruption of cellular redox homeostasis, excitotoxicity, and activation of inflammatory signaling pathways [2,3]. These mechanisms collectively contribute to neuronal degeneration, vascular dysfunction, white matter injury, and long-term neurological deficits [4].

Experimental studies investigating cerebral I/R injury have traditionally focused on the cerebral cortex, hippocampus, and striatum because these regions are directly affected by middle cerebral artery occlusion (MCAO) [5]. However, increasing evidence suggests that supratentorial ischemic injury may also induce secondary alterations in remote brain regions, particularly the cerebellum, through disrupted cerebro-cerebellar connectivity, inflammatory propagation, oxidative stress, and metabolic dysfunction [6,7]. This phenomenon, frequently described as crossed cerebellar diaschisis or secondary cerebellar degeneration, has been associated with impaired motor coordination, gait abnormalities, and functional neurological deterioration following ischemic stroke [7,8]. Therefore, evaluation of cerebellar involvement may provide additional insight into the systemic and remote neuroinflammatory consequences of cerebral ischemia.

The cerebellum appears particularly vulnerable to oxidative and inflammatory injury because of its high metabolic activity, dense synaptic organization, and relatively limited intrinsic antioxidant capacity [9]. Purkinje cells, which represent the principal output neurons of the cerebellar cortex, are among the most ischemia-sensitive neuronal populations in the central nervous system [10,11]. Experimental studies have shown that oxidative stress and inflammatory cytokines promote Purkinje cell degeneration through mitochondrial dysfunction, calcium dysregulation, excitotoxicity, and apoptotic signaling pathways [11]. In parallel, ischemia-associated inflammatory activation contributes to cerebellar white matter injury and myelin degeneration through microglial activation, endothelial dysfunction, cytokine release, and oligodendrocyte damage [12].

Neuroinflammation plays a central role in the progression of ischemia–reperfusion injury. Following cerebral ischemia, activated microglia release pro-inflammatory cytokines and reactive oxygen species that amplify secondary neuronal and vascular injury [13,14]. Among these mediators, tumor necrosis factor-alpha (TNF-α) is particularly important because it contributes to endothelial activation, blood–brain barrier disruption, leukocyte recruitment, and apoptotic signaling [15]. Sustained TNF-α signaling has been closely associated with increased neuronal loss, demyelination, and poor neurological outcome after ischemic stroke [16]. In addition, activated microglia and TNF-α-mediated inflammatory pathways have been implicated in oligodendrocyte dysfunction and myelin degeneration during neuroinflammatory conditions [17]. Therefore, simultaneous suppression of oxidative stress and neuroinflammation may represent an effective strategy for limiting secondary cerebellar injury following cerebral ischemia.

Natural alkaloids derived from medicinal plants have attracted increasing attention because of their antioxidant, anti-inflammatory, and neuroprotective properties [18]. Skimmianine, a furoquinoline alkaloid isolated from plants of the Rutaceae family, has been reported to exhibit antioxidant, anti-inflammatory, anticonvulsant, and neuromodulatory effects [19]. Previous experimental studies demonstrated that skimmianine suppresses inflammatory cytokine production, reduces ROS-mediated tissue injury, and attenuates ischemia-associated oxidative stress [20,21,22]. Moreover, recent evidence suggests that skimmianine may exert neuroprotective effects in experimental cerebral ischemia through modulation of inflammatory and oxidative signaling pathways [23]. However, its potential protective effects on secondary cerebellar alterations, microglial activation, and myelin ultrastructural injury following cerebral ischemia–reperfusion remain insufficiently characterized.

Therefore, the present study aimed to investigate the protective effects of skimmianine pretreatment on cerebellar oxidative stress, neuroinflammation, Purkinje cell degeneration, microglial activation, and myelin ultrastructural alterations following experimental cerebral ischemia–reperfusion injury. In addition to biochemical, histopathological, immunohistochemical, behavioral, and ultrastructural analyses, in silico network pharmacology and molecular docking approaches were employed to further explore the potential TNF-α-associated molecular pathways modulated by skimmianine.

## 2. Materials and Methods

### 2.1. Experimental Design

This study was conducted in accordance with the approval of the Animal Experiments Local Ethics Committee of Dicle University (Approval Number: 2025/07, approval date: 27 November 2025). A total of 32 female Wistar albino rats, aged 10–12 weeks and weighing 240–260 g, were used. The animals were randomly divided into four experimental groups (*n* = 8 per group). Rats were housed under controlled conditions with a 12 h light/dark cycle (08:00–20:00), at a temperature of 23 ± 2 °C, with ad libitum access to standard pellet diet and water. Skimmianine (catalog no: HY-N2081, MedChemExpress, Shanghai, China) and 0.5% sodium carboxymethyl cellulose solution (CAS 9004-32-4, Santa Cruz Biotechnology Inc., Dallas, TX, USA) were used as the vehicle and experimental compound.

### 2.2. Induction of Cerebral I/R and Experimental Groups

Rats were anesthetized intramuscularly with ketamine hydrochloride (90 mg/kg, Ketalar, Pfizer, Istanbul, Turkey) and xylazine (15 mg/kg, Rompun, Bayer, Istanbul, Turkey). Animals were placed in a supine position, and the neck region was disinfected with povidone-iodine. A midline incision of approximately 3 cm was made from the upper edge of the sternum to the hyoid bone. The incision was widened using a tissue retractor, and the trachea was exposed by blunt dissection. Paratracheal muscles were carefully dissected to reach the common carotid artery (CCA). The internal carotid artery lumen was accessed using a 4-0 monofilament nylon suture, which was advanced to conduct MCAO. Ischemia was maintained for 60 min, after which the suture was removed, and the incision was closed. A reperfusion period of 23 h was established. The selected skimmianine dose (40 mg/kg) was based on our previous experimental study, in which this dose demonstrated significant neuroprotective efficacy against cerebral ischemia/reperfusion injury without observable adverse effects [21]. Experimental groups:

1. Sham group: The left CCA was isolated without inducing ischemia. Rats received intraperitoneal injections of 1 cc of 0.5% sodium carboxymethyl cellulose solution daily for 14 days.
2. I/R group: Cerebral I/R injury was induced as described. Rats received intraperitoneal 1 cc 0.5% sodium carboxymethyl cellulose solution daily for 14 days prior to ischemia.
3. Skimmianine group: No cerebral I/R was performed. Rats received intraperitoneal Skimmianine (40 mg/kg) dissolved in 1 cc 0.5% sodium carboxymethyl cellulose daily for 14 days.
4. I/R + Skimmianine group: Cerebral I/R was induced. Rats received intraperitoneal Skimmianine (40 mg/kg) dissolved in 1 cc 0.5% sodium carboxymethyl cellulose daily for 14 days prior to ischemia.

At the end of the reperfusion period, animals were euthanized under general anesthesia by exsanguination. Blood samples were collected for biochemical analyses, and cerebellar tissues were dissected and fixed for histological and immunohistochemical evaluation.

### 2.3. Behavioral Assessment of Cerebellar Motor Coordination

Cerebellar motor coordination and sensorimotor deficits were evaluated using beam walking and grid walking tests before euthanasia. All behavioral assessments were performed by an observer blinded to the experimental groups. The beam walking test was performed using a modified protocol based on previously described methods [24,25]. Rats were placed on one end of a narrow elevated beam and allowed to traverse toward a dark goal box. Before testing, animals were habituated to the apparatus. During the test session, the time required to cross the beam and the number of hindlimb foot slips were recorded. Increased crossing time and higher foot-slip numbers were considered indicators of impaired motor coordination and balance. The grid walking test was conducted using a modified version of a previously established method [26]. Sensorimotor coordination was assessed using a horizontal wire grid apparatus. Each rat was allowed to freely walk on the grid for a fixed observation period. The total number of steps and the number of foot faults, defined as complete paw slips through the grid openings, were recorded. The foot fault percentage was calculated as follows: Foot fault (%) = number of foot faults/total number of steps × 100. Higher foot fault percentages indicated greater sensorimotor impairment. Behavioral data were compared among groups and correlated with histopathological and inflammatory parameters where appropriate.

### 2.4. Determination of Total Antioxidant Status (TAS) and Total Oxidant Status (TOS)

TAS and TOS levels were measured to evaluate the systemic oxidative balance in experimental groups. Blood samples were centrifuged at 3000 rpm for 10 min at 4 °C, and the obtained serum was aliquoted and stored at −80 °C until analysis. Serum TAS and TOS levels were determined using commercially available colorimetric assay kits (catalog no: RL0017, catalog no: RL0024, Rel Assay Diagnostics, Gaziantep, Turkey, respectively), in accordance with the manufacturer’s instructions. Biochemical analyses were performed using an automated chemistry analyzer (AU5800; Beckman Coulter Inc., Brea, CA, USA) with an absorbance measurement spectrophotometrically at a wavelength of 532 nm. TAS measurements were based on the ability of antioxidants in the sample to suppress the formation of the colored 2,2′-azinobis-(3-ethylbenzothiazoline-6-sulfonate) radical cation (ABTS•^+^). The change in absorbance was measured spectrophotometrically, and results were expressed as mmol Trolox equivalents per liter (mmol Trolox Eq/L) [27]. TOS measurements were performed using a method based on the oxidation of ferrous ion to ferric ion in the presence of oxidant molecules in the sample. The ferric ion forms a colored complex with xylenol orange in an acidic medium, and the intensity of the color is directly proportional to the total oxidant molecules present in the sample. Absorbance was measured spectrophotometrically, and TOS values were expressed as µmol hydrogen peroxide equivalents per liter (µmol H_2_O_2_ Eq/L) [28]. All measurements were performed in duplicate, and the mean values were used for statistical analysis. The assays demonstrated high intra- and inter-assay precision, as reported by the manufacturer.

### 2.5. Determination of Serum TNF-α, GFAP, and Iba1 Levels Measurements

Serum TNF-α, glial fibrillary acidic protein (GFAP), and ionized calcium-binding adapter molecule 1 (Iba1/AIF-1) levels were quantitatively determined using commercially available rat-specific enzyme-linked immunosorbent assay (ELISA) kits according to the manufacturers’ instructions. TNF-α concentrations were measured using a Rat TNF-α ELISA Kit (Elabscience Biotechnology, Houston, TX, USA; Cat. No: E-CL-R0019), GFAP concentrations using a Rat GFAP ELISA Kit (Elabscience Biotechnology, Houston, TX, USA; Cat. No: E-EL-R1428), and Iba1 concentrations using a Rat AIF-1/Iba1 ELISA Kit (Novus Biologicals, Centennial, CO, USA; Cat. No: NBP2-66675). The reported assay sensitivities were 2.7 pg/mL for TNF-α, 0.19 ng/mL for GFAP, and 18.75 pg/mL for Iba1. Blood samples were collected by cardiac puncture at the end of the experimental period and centrifuged at 3000 rpm for 10 min at 4 °C. The obtained serum samples were aliquoted and stored at −80 °C until analysis. Before assay, samples were thawed at room temperature and gently mixed. For each assay, 100 μL of standards and serum samples were added to antibody-coated microplate wells and incubated for 90 min at 37 °C. After removal of the liquid, biotinylated detection antibody was added and incubated for 60 min at 37 °C, followed by incubation with horseradish peroxidase (HRP)-conjugated streptavidin for 30 min at 37 °C. After the washing procedures, tetramethylbenzidine (TMB) substrate solution was added and allowed to react for 15 min in the dark. The reaction was terminated using stop solution, and absorbance was measured at 450 nm using a microplate reader (BioTek ELx800, BioTek Instruments, Winooski, VT, USA). Concentrations of TNF-α, GFAP, and Iba1 were calculated from standard curves generated using recombinant standards supplied by the manufacturers and expressed as pg/mL or ng/mL as appropriate. All samples were analyzed in duplicate, and the mean values were used for statistical analysis. The intra-assay and inter-assay coefficients of variation were below 10% for all ELISA kits.

### 2.6. Histological Tissue Processing

Cerebellar tissues were fixed in 10% formalin for 24 h. Tissues were dehydrated through graded alcohol series (70%, 80%, 90%, 96%), cleared in xylene, and embedded in paraffin. Sections of 4–5 µm thickness were obtained using a microtome and mounted on adhesive slides. Sections were deparaffinized at 58–62 °C for 6 h, rehydrated, and stained with Hematoxylin and Eosin to evaluate general histopathological changes such as hemorrhage, inflammation, and congestion/dilation. Cerebellar sections were semi-quantitatively evaluated under light microscopy by an experienced histologist blinded to the experimental groups. The following parameters were scored separately in the cerebellar cortex and white matter: Purkinje cell loss, edema (molecular and granular layers) and vascular congestion. Each parameter was scored using a four-point scale: 0 = none (normal histology), 1 = mild, 2 = moderate and 3 = severe [29]. For each animal, ten randomly selected fields were evaluated at ×200 magnification, and mean scores were calculated for statistical analysis. TNF-α immunoreactivity in cerebellar sections was evaluated semi-quantitatively by a blinded histologist using light microscopy.

### 2.7. Immunohistochemical Analysis

Immunohistochemical staining was carried out to evaluate TNF-α expression in cerebellar tissue sections using a streptavidin–biotin–peroxidase method. Formalin-fixed, paraffin-embedded cerebellar tissue samples were cut into 4–5 μm sections and placed on poly-L-lysine-coated slides. After deparaffinization in xylene, the sections were rehydrated through descending ethanol concentrations to distilled water. Antigen retrieval was achieved in EDTA buffer (pH 8.0; catalog no: ab93680, Abcam, Cambridge, MA, USA) using microwave heating for 15 min. Once cooled to room temperature, endogenous peroxidase activity was inhibited with 3% hydrogen peroxide solution (catalogue no: TA-015-HP, Thermo Fisher Scientific, Waltham, MA, USA) for 10 min. Nonspecific background staining was minimized by incubating the sections with a protein blocking reagent (catalogue no: TA-125-UB, Thermo Scientific, Waltham, MA, USA) for 15 min at room temperature. The sections were then exposed overnight at 4 °C to the primary TNF-α antibody (catalogue no: sc-52746, Santa Cruz Biotechnology, Inc., Dallas, TX, USA; dilution 1:200). After rinsing with phosphate-buffered saline, the sections were treated with a biotinylated secondary antibody (catalogue no: TP-125-BN, Thermo Scientific, Waltham, MA, USA) for 30 min at room temperature, followed by incubation with streptavidin–peroxidase complex (catalogue no: TS-125-HR, Thermo Scientific, Waltham, MA, USA) for an additional 30 min. The antigen–antibody reaction was visualized with 3,3-diaminobenzidine (DAB; catalogue no: TA-125-HD, Thermo Scientific, Waltham, MA, USA), resulting in a brown staining pattern. Images were captured using a Zeiss Imager A2 photomicroscope (Carl Zeiss Microscopy GmbH, Jena, Germany). Staining intensity and distribution were assessed separately in the molecular layer (m), granular layer (g) and Purkinje cell layer. Immunoreactivity was scored according to the following scale: 0 = negative, 1 = weak, 2 = moderate, 3 = strong [29]. For each animal, ten randomly selected fields were analyzed at ×200 magnification, and mean scores were calculated for statistical evaluation.

### 2.8. Ultrastructural Transmission Electron Microscopy Analysis

Cerebellar tissues were fixed in 2.5% glutaraldehyde at 4 °C for 24 h and subsequently post-fixed in Karnovsky’s fixative overnight at 4 °C. The samples were rinsed twice with cacodylate buffer for 45 min each and kept in the same buffer overnight at 4 °C. Thereafter, the tissues were fixed in 1% osmium tetroxide for 2 h at 4 °C in the dark and rinsed twice with cacodylate buffer for 15 min each. The specimens were dehydrated through a graded ethanol series consisting of 25% and 50% ethanol for 10 min each, followed by overnight incubation in 75% ethanol at 4 °C. On the following day, the tissues were incubated in 2% uranyl acetate prepared in 75% ethanol for 45 min at room temperature. Further dehydration was performed in 90% and 100% ethanol for 10 min each at 4 °C, followed by an additional 30 min in 100% ethanol at room temperature. The samples were then treated twice with propylene oxide for 10 min and incubated in a 1:1 mixture of propylene oxide and embedding resin at 25 °C for 15 min. Subsequently, the tissues were immersed twice in pure resin, embedded in capsules, and polymerized at 45 °C overnight and at 60 °C for 24 h. Ultra-thin sections (70 nm) were then cut and mounted on copper grids. The sections were stained with 2% uranyl acetate for 30 min, rinsed with distilled water, and counterstained with lead citrate for 10 min. Ultrastructural examination was performed using a JEOL JEM-1010 transmission electron microscope (JEOL Ltd., Tokyo, Japan). All TEM analyses were conducted on coded samples, and the evaluator was blinded to the treatment groups during the assessment process.

### 2.9. Quantitative Ultrastructural Analysis of Myelin Integrity

Quantitative ultrastructural analysis was performed on transmission electron microscopy images to evaluate myelin sheath integrity in cerebellar white matter. For each animal, randomly selected myelinated nerve fibers with clearly identifiable axonal and myelin boundaries were analyzed at high magnification. Measurements were performed using ImageJ software (version 1.54t) (National Institutes of Health, Bethesda, MD, USA) by an investigator blinded to the experimental groups. Axon diameter was defined as the inner diameter of the myelinated fiber excluding the myelin sheath, whereas fiber diameter represented the total outer diameter including the myelin sheath. Myelin thickness was calculated using the following formula: Myelin thickness = Fiber diameter − Axon diameter/2.

The g-ratio was calculated as the ratio of axon diameter to total fiber diameter using the following formula: g-ratio = axon diameter/fiber diameter. In addition, vacuolized fibers were quantified and expressed as the percentage of myelinated fibers exhibiting vacuolar degeneration or lamellar separation. A total of at least 100 myelinated fibers per group were analyzed for quantitative evaluation [30].

### 2.10. In Silico Network Pharmacology and Functional Enrichment Analysis

To investigate the potential biological responses mediated by TNF-α that may be modulated by skimmianine, an in silico analysis was performed. Skimmianine-related protein targets were retrieved from the ChEMBL and SwissTargetPrediction databases. In parallel, TNF-α–associated protein targets were obtained from the STRING database by selecting 100 additional interactions with a medium confidence level (0.400) [31,32]. The protein target sets derived from skimmianine and TNF-α were imported into Cytoscape (version 3.10.4), where an intersection analysis was performed to identify shared proteins. Gene Ontology Biological Process (GO BP) enrichment analysis of the intersecting proteins was conducted using the ShinyGO (version 0.85.1). Pathways with a false discovery rate (FDR) < 0.05 were considered statistically significant, and the top 10 enriched pathways were ranked in descending order based on fold enrichment.

### 2.11. Molecular Docking Analysis

Molecular docking analysis was performed to investigate the potential interaction between skimmianine and TNF-α. The three-dimensional structure of skimmianine was obtained from the PubChem database (PubChem CID: 6760). The crystal structure of TNF-α was retrieved from the Protein Data Bank (PDB ID: 2AZ5). Prior to docking, the protein structure was prepared by removing unnecessary heteroatoms. Docking simulations were carried out using AutoDock Vina (Version 1.54t). The docking grid center was defined according to the geometric center of the TNF-α structure at x = −13.6764, y = 71.5974, and z = 26.9904. Grid box dimensions were set to 25 × 25 × 25 Å to ensure coverage of the putative binding region. Default docking parameters were used, and multiple binding poses were generated. The most favorable binding conformation was selected based on the lowest binding energy value. Protein–ligand interactions and residue proximity analyses were visualized using PyMOL Molecular Graphics System (version 3.1.8, Schrödinger LLC, New York, NY, USA) [33].

### 2.12. Statistical Analysis

Statistical analyses were performed using IBM SPSS Statistics version 25.0 (IBM Corp., Armonk, NY, USA). Data were presented as mean ± standard deviation (SD). The normality of data distribution was evaluated using the Shapiro–Wilk test. Comparisons among the four experimental groups were performed using one-way analysis of variance (ANOVA) followed by Tukey’s post hoc test for pairwise comparisons. Although histopathological and immunohistochemical scores originated from ordinal scales, statistical analyses were performed using parametric methods because the final values represented mean scores obtained from multiple microscopic fields per animal. Normality was assessed using the Shapiro–Wilk test, and no substantial deviations from normal distribution were detected. Therefore, one-way ANOVA followed by Tukey’s post hoc test was considered appropriate for group comparisons. Correlation analysis between serum TNF-α levels and cerebellar TNF-α immunoreactivity scores was performed using Spearman rank correlation analysis. Correlation coefficients (r) were interpreted according to the strength and direction of association. A *p*-value < 0.05 was considered statistically significant.

## 3. Results

### 3.1. Skimmianine Improved Cerebellar Motor Coordination and Sensorimotor Function Following Cerebral I/R Injury

Behavioral assessment revealed significant differences among the experimental groups in all measured parameters (Table 1). One-way ANOVA demonstrated significant group effects for beam crossing time [F(3,28) = 39.52, *p* < 0.001], beam foot slips [F(3,28) = 84.48, *p* < 0.001], grid total steps [F(3,28) = 12.61, *p* < 0.001], grid foot faults [F(3,28) = 50.78, *p* < 0.001], and grid foot fault rate [F(3,28) = 58.44, *p* < 0.001]. The sham and skimmianine groups exhibited comparable behavioral performance, indicating that skimmianine administration alone did not adversely affect motor coordination or sensorimotor function. In contrast, animals subjected to cerebral I/R injury displayed marked motor impairment, characterized by prolonged beam crossing times, increased numbers of beam foot slips and grid foot faults, reduced total grid steps, and a significantly higher foot fault rate compared with the sham group (*p* < 0.05). Treatment with skimmianine significantly ameliorated these behavioral deficits. Animals in the I/R + Skimmianine group crossed the beam more rapidly, exhibited fewer foot slips and foot faults, and demonstrated a lower foot fault rate than untreated I/R animals (*p* < 0.05). In addition, total grid steps were significantly increased following skimmianine treatment. Although behavioral performance did not completely return to sham levels, these findings indicate that skimmianine improved early behavioral performance in beam walking and grid walking tests following I/R injury following I/R injury.

### 3.2. Skimmianine Restored Antioxidant Capacity and Reduced Oxidative Stress Following Cerebral I/R

Serum oxidative stress parameters differed significantly among the experimental groups (Table 2). One-way ANOVA revealed significant group effects for TAS [F(3,28) = 24.91, *p* < 0.001], TOS [F(3,28) = 31.92, *p* < 0.001], and OSI [F(3,28) = 1549.58, *p* < 0.001]. The I/R group exhibited a marked decrease in TAS levels together with substantial increases in TOS and OSI values compared with the sham group (*p* < 0.05), indicating severe oxidative stress following cerebral I/R injury. In contrast, animals treated with skimmianine showed significantly higher TAS levels and lower TOS and OSI values than untreated I/R animals (*p* < 0.05). Although behavioral performance did not completely return to sham levels, these findings indicate that skimmianine improved early sensorimotor performance following I/R injury.

### 3.3. Skimmianine Attenuated Neuroinflammatory Responses Following Cerebral I/R Injury

Serum TNF-α, Iba1, and GFAP levels differed significantly among the experimental groups (Table 3). One-way ANOVA revealed significant group effects for TNF-α [F(3,28) = 84.52, *p* < 0.001], Iba1 [F(3,28) = 38.75, *p* < 0.001], and GFAP [F(3,28) = 686.83, *p* < 0.001]. Baseline concentrations of all three biomarkers were low in the sham and skimmianine groups. In contrast, cerebral I/R injury markedly increased serum TNF-α, Iba1, and GFAP levels in the I/R group compared with sham (*p* < 0.05). Skimmianine treatment significantly attenuated these elevations, resulting in lower TNF-α, Iba1, and GFAP levels in the I/R + Skimmianine group compared with the untreated I/R group (*p* < 0.05). Although biomarker levels remained higher than those observed in the sham group, the substantial reductions in TNF-α, Iba1, and GFAP suggest that skimmianine mitigated the systemic neuroinflammatory response and reduced markers associated with glial injury following ischemia/reperfusion.

### 3.4. Skimmianine Preserved Cerebellar Histoarchitecture in H&E-Stained Sections

**Figure 1:**
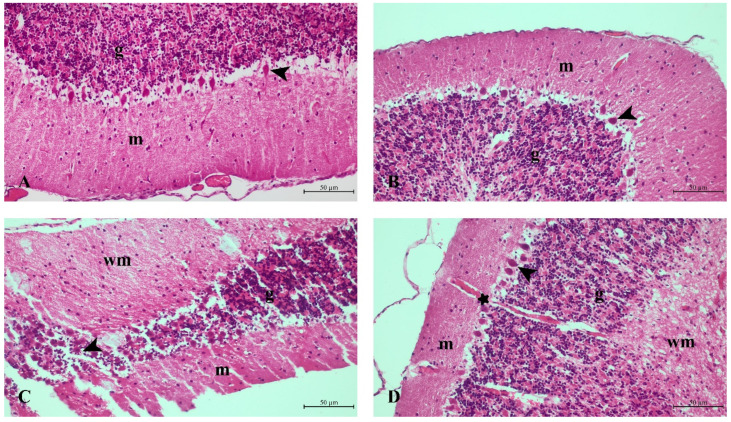
Representative Hematoxylin and Eosin-stained cerebellar sections from the (**A**), Sham, (**B**) Skimmianine, (**C**) I/R, and (**D**) I/R + Skimmianine groups. g, granular layer; m, molecular layer; wm, white matter; arrow, Purkinje cell layer; asterisk, vascular congestion. Scale bar = 50 μm.

Representative Hematoxylin and Eosin-stained cerebellar sections are shown in Figure 1. The sham and skimmianine groups exhibited normal cerebellar histoarchitecture with well-organized molecular and granular layers and an intact Purkinje cell layer. No evidence of edema, vascular congestion, or neuronal degeneration was observed (Figure 1A,B). In contrast, the I/R group demonstrated marked histopathological alterations, including Purkinje cell loss, cellular degeneration, edema, disruption of cortical organization, and vascular congestion. Structural disorganization was also evident within the white matter, indicating substantial I/R-induced cerebellar injury (Figure 1C). Treatment with skimmianine markedly attenuated these alterations. The I/R + Skimmianine group showed improved preservation of cerebellar architecture, reduced edema and vascular congestion, and a greater number of morphologically intact Purkinje cells compared with the untreated I/R group. Although mild abnormalities persisted, overall tissue integrity was substantially improved following skimmianine treatment (Figure 1D).

### 3.5. Skimmianine Suppressed Cerebellar TNF-α Immunoreactivity After Cerebral I/R

**Figure 2:**
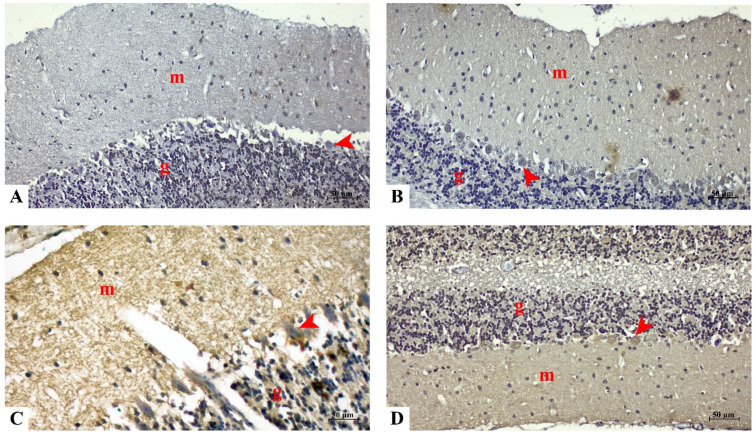
Representative TNF-α-immunostained cerebellar sections from the (**A**) Sham, (**B**) Skimmianine, (**C**) I/R, and (**D**) I/R + Skimmianine groups. g, granular layer; m, molecular layer; arrow, Purkinje cell layer; Scale bar = 50 μm.

Representative TNF-α immunohistochemical staining findings in cerebellar tissue are shown in Figure 2. The sham and skimmianine groups exhibited weak or absent TNF-α immunoreactivity in the molecular and granular layers, with no apparent staining in the Purkinje cell layer (Figure 2A,B). In contrast, the I/R group demonstrated markedly increased TNF-α expression throughout the cerebellar cortex, particularly in the molecular and granular layers, Purkinje cell layer, and vascular structures, indicating pronounced inflammatory activation following I/R injury (Figure 2C). Skimmianine treatment substantially reduced TNF-α immunoreactivity in the I/R + Skimmianine group, although mild positivity remained in some regions (Figure 2D). These findings suggest that skimmianine attenuated TNF-α-mediated inflammatory responses in cerebellar tissue following I/R injury.

### 3.6. Semi-Quantitative Analysis Confirmed the Protective Effects of Skimmianine on Cerebellar Histopathology and TNF-α Expression

Semi-quantitative analysis demonstrated significant differences among the experimental groups for Purkinje cell loss, edema, vascular congestion, and TNF-α expression (Table 4). One-way ANOVA revealed significant group effects for Purkinje cell loss [F(3,28) = 98.87, *p* < 0.001], edema [F(3,28) = 58.71, *p* < 0.001], vascular congestion [F(3,28) = 54.17, *p* < 0.001], and TNF-α expression [F(3,28) = 26.70, *p* < 0.001]. The sham and skimmianine groups exhibited minimal histopathological alterations, characterized by negligible Purkinje cell loss, mild edema, absence of vascular congestion, and weak TNF-α immunoreactivity. No significant differences were observed between these groups. In contrast, the I/R group showed marked cerebellar injury, including substantial Purkinje cell loss, pronounced edema, severe vascular congestion, and significantly increased TNF-α expression compared with the sham group (*p* < 0.05). These findings indicate extensive tissue damage and inflammatory activation following cerebral I/R injury. Skimmianine treatment significantly ameliorated these alterations in the I/R + Skimmianine group. Histopathological scores and TNF-α expression were significantly lower than those observed in the untreated I/R group (*p* < 0.05). Although mild abnormalities persisted, the reductions in Purkinje cell loss, edema, vascular congestion, and TNF-α immunoreactivity suggest that skimmianine attenuated I/R-induced cerebellar injury and inflammatory responses.

Quantitative morphometric analysis demonstrated significant differences among the experimental groups in Purkinje cell count, soma area, and Purkinje layer thickness (Table 5). One-way ANOVA revealed significant group effects for Purkinje cell count [F(3,28) = 64.07, *p* < 0.001], Purkinje cell soma area [F(3,28) = 61.88, *p* < 0.001], and Purkinje layer thickness [F(3,28) = 43.66, *p* < 0.001]. The I/R group exhibited a marked reduction in Purkinje cell density, soma area, and layer thickness compared with the sham and skimmianine groups (*p* < 0.05). In contrast, skimmianine treatment significantly ameliorated these alterations, resulting in higher Purkinje cell counts, larger soma areas, and greater Purkinje layer thickness in the I/R + Skimmianine group compared with the untreated I/R group (*p* < 0.05). However, all morphometric parameters remained below sham values, indicating partial but significant structural recovery following skimmianine administration.

### 3.7. Serum TNF-α Levels Were Positively Correlated with Cerebellar TNF-α Immunoreactivity

To evaluate the relationship between systemic inflammation and local cerebellar inflammatory response, correlation analysis was performed between serum TNF-α levels measured by ELISA and semi-quantitative TNF-α immunohistochemical scores in cerebellar tissue. A strong positive correlation was observed between serum TNF-α concentrations and cerebellar TNF-α immunoreactivity scores (r = 0.74, *p* < 0.001). Animals exhibiting higher circulating TNF-α levels also demonstrated increased TNF-α expression in the molecular and granular layers, as well as in the Purkinje cell layer. Conversely, reduced serum TNF-α levels in the I/R + Skimmianine group were accompanied by attenuated tissue TNF-α immunoreactivity. These findings indicate that systemic TNF-α elevation following ischemia–reperfusion is closely associated with local cerebellar inflammatory activation and that skimmianine-mediated suppression of serum TNF-α levels is paralleled by reduced cerebellar TNF-α expression. This concordance between biochemical and immunohistochemical data strengthens the evidence for the anti-inflammatory effect of skimmianine in cerebellar tissue.

### 3.8. Skimmianine Preserved Myelin Sheath Ultrastructure Following Cerebral I/R

**Figure 3:**
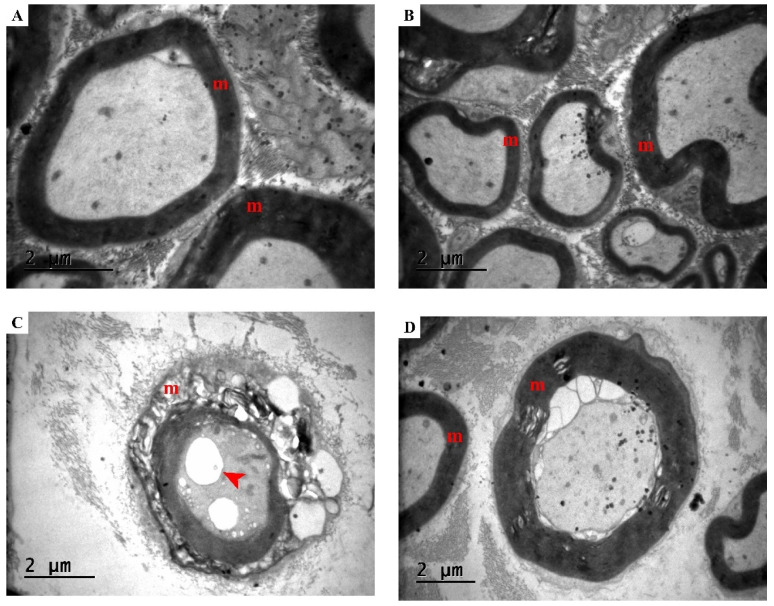
Representative transmission electron microscopy images from the (**A**) Sham, (**B**) Skimmianine, (**C**) I/R, and (**D**) I/R + Skimmianine groups. m: myelin sheath, arrow: vacuole, Scale bar = 2 µm.

Representative transmission electron microscopy findings are shown in Figure 3. The sham and skimmianine groups exhibited well-preserved myelinated nerve fibers with compact and regularly organized myelin lamellae, without evidence of significant ultrastructural abnormalities (Figure 3A,B). In contrast, the I/R group demonstrated marked myelin damage characterized by vacuolization, separation of myelin lamellae, disruption of sheath integrity, and loss of compact myelin organization (Figure 3C). Skimmianine treatment markedly attenuated these alterations, resulting in improved preservation of myelin structure, reduced vacuolization, and more compact myelin lamellae compared with the untreated I/R group (Figure 3D). Although mild ultrastructural abnormalities persisted, overall myelin integrity was substantially improved following skimmianine treatment.

### 3.9. Skimmianine Preserved Myelin Integrity and Reduced Ultrastructural Damage Following Cerebral I/R Injury

Quantitative ultrastructural analysis demonstrated significant differences among the experimental groups in fiber diameter, myelin thickness, G-ratio, and the percentage of vacuolized fibers (Table 6). One-way ANOVA revealed significant group effects for fiber diameter [F(3,28) = 8.31, *p* < 0.001], myelin thickness [F(3,28) = 34.72, *p* < 0.001], G-ratio [F(3,28) = 14.85, *p* < 0.001], and vacuolized fibers [F(3,28) = 123.51, *p* < 0.001]. In contrast, axon diameter did not differ significantly among the groups [F(3,28) = 0.39, *p* = 0.760]. The I/R group exhibited marked ultrastructural deterioration characterized by reduced fiber diameter, pronounced myelin thinning, increased G-ratio values, and a substantial increase in vacuolized fibers compared with the sham group (*p* < 0.05). These findings indicate severe myelin disruption and axonal degeneration following I/R injury. Skimmianine treatment significantly ameliorated these alterations. Animals in the I/R + Skimmianine group exhibited greater fiber diameter and myelin thickness, lower G-ratio values, and fewer vacuolized fibers than those in the untreated I/R group (*p* < 0.05). Although these parameters did not completely return to sham levels, the observed improvements indicate that skimmianine partially preserved myelin integrity and attenuated ultrastructural damage induced by I/R.

### 3.10. Shared Molecular Targets Suggested That Skimmianine Modulates TNF-α-Associated Inflammatory Pathways

**Figure 4:**
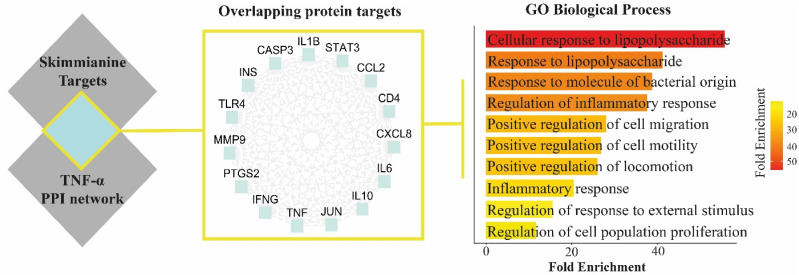
Network pharmacology and GO Biological Process enrichment analysis of skimmianine-associated TNF-α signaling. The middle panel shows the protein–protein interaction network of the 15 overlapping targets, whereas the right panel displays the top 10 significantly enriched GO Biological Process terms. Colors indicate fold enrichment from red (high) to yellow (low).

The results of the in silico network pharmacology and GO enrichment analyses are presented in Figure 4. Integration of skimmianine-associated targets with the TNF-α protein–protein interaction network identified 15 overlapping proteins, suggesting potential molecular interactions between skimmianine and TNF-α-related signaling pathways. The shared target network included key inflammatory mediators such as TNF, IL6, IL1B, TLR4, STAT3, IFNG, and IL10. Gene Ontology biological process enrichment analysis revealed significant enrichment of inflammation- and stimulus-responsive pathways (FDR < 0.05). The most enriched biological processes included cellular response to lipopolysaccharide, response to molecules of bacterial origin, regulation of inflammatory response, inflammatory response, regulation of response to external stimulus, positive regulation of cell migration, and regulation of cell population proliferation (Figure 4). These findings suggest that skimmianine may exert anti-inflammatory effects through modulation of TNF-α-associated signaling networks.

### 3.11. Molecular Docking Demonstrated a Favorable Interaction Between Skimmianine and TNF-α

**Figure 5:**
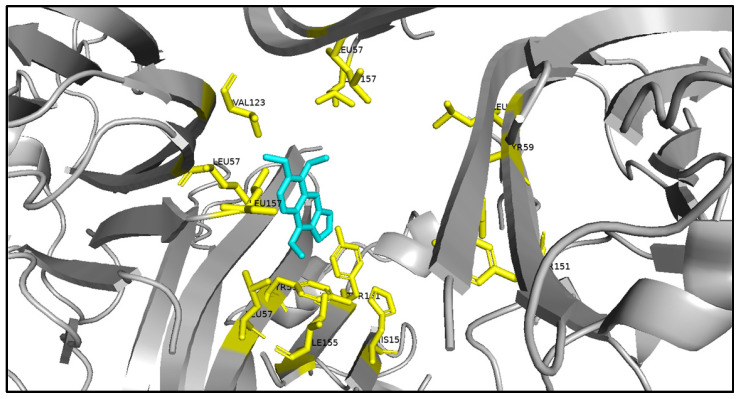
Molecular docking analysis of skimmianine with TNF-α. The predicted binding pose of skimmianine within the TNF-α binding cavity is shown. Skimmianine is displayed in green, while the TNF-α protein structure is shown in gray. Surrounding amino acid residues involved in ligand stabilization are displayed and labeled. The docking simulation yielded a binding energy of −6.953 kcal/mol, indicating a favorable ligand–protein interaction.

The predicted molecular interaction between skimmianine and TNF-α is presented in Figure 5. Molecular docking analysis demonstrated a favorable binding affinity of skimmianine toward TNF-α, with a predicted binding energy of −6.953 kcal/mol. The best-ranked docking pose positioned skimmianine within a surface-accessible binding cavity of TNF-α, where it interacted with several surrounding residues, including Tyr59, Tyr119, Tyr151, Leu55, Leu57, Leu58, Leu157, Val123, and His15. The binding pocket was predominantly hydrophobic and exhibited good spatial complementarity with the ligand. These findings suggest a potential molecular interaction between skimmianine and TNF-α; however, the functional significance of this interaction remains to be experimentally validated.

## 4. Discussion

The present study demonstrates that cerebral ischemia–reperfusion injury is associated with substantial secondary cerebellar alterations involving oxidative stress, neuroinflammation, Purkinje cell degeneration, white matter injury, and functional motor impairment. Although MCAO primarily affects supratentorial structures, increasing evidence indicates that ischemic injury may trigger remote pathological responses in anatomically connected brain regions through inflammatory propagation, disrupted neuronal connectivity, and metabolic dysregulation [34,35]. The current findings support this concept by showing that cerebral ischemia was accompanied by marked cerebellar histopathological, ultrastructural, inflammatory, and behavioral abnormalities. More importantly, skimmianine pretreatment attenuated these alterations, suggesting that modulation of oxidative and inflammatory pathways may limit secondary cerebellar injury following cerebral ischemia.

Oxidative stress is recognized as one of the earliest and most important mechanisms contributing to ischemia–reperfusion injury [36]. Excessive reactive oxygen species generation during reperfusion promotes lipid peroxidation, mitochondrial dysfunction, DNA damage, and activation of downstream inflammatory cascades [37]. In the present study, cerebral ischemia resulted in a marked disruption of systemic oxidant–antioxidant balance, characterized by reduced TAS levels and increased TOS and OSI values. These findings are consistent with previous reports demonstrating enhanced oxidative burden following cerebral ischemia and support the concept that oxidative stress is not restricted to the infarcted territory but may contribute to injury in remote brain regions, including the cerebellum [7,11]. The attenuation of oxidative stress markers by skimmianine is in agreement with previous experimental studies showing antioxidant properties of this furoquinoline alkaloid in different pathological conditions and stress [20,38]. Therefore, restoration of redox homeostasis may represent one of the principal mechanisms underlying the neuroprotective effects observed in the present study.

Accumulating evidence indicates that neuroinflammation represents a major determinant of secondary injury progression after ischemic stroke. Activated microglia rapidly respond to ischemic signals and amplify tissue damage through the production of pro-inflammatory cytokines, reactive oxygen species, and neurotoxic mediators [16]. The present study extends previous observations by demonstrating not only increased TNF-α levels but also elevated Iba1 and GFAP concentrations following cerebral I/R. These findings suggest a robust systemic neuroinflammatory response accompanied by increased circulating biomarkers associated with glial injury and immune activation following cerebral I/R. Importantly, skimmianine treatment significantly reduced all three inflammatory markers. This observation is particularly noteworthy because a recent in vitro study demonstrated that skimmianine suppresses inflammatory responses in lipopolysaccharide-stimulated BV-2 microglial cells and reduces the production of pro-inflammatory mediators [39,39]. Together with the current in vivo findings, these data suggest that modulation of inflammation-associated pathways may constitute an important mechanism through which skimmianine limits neuroinflammatory damage. The concomitant reduction in TNF-α further supports this hypothesis, as TNF-α is a major downstream effector of activated microglia and has been implicated in blood–brain barrier dysfunction, neuronal injury, and demyelination after stroke [40,41,42].

An additional strength of the present study is the integration of structural and functional outcome measures. Purkinje cells are among the most vulnerable neuronal populations in the cerebellum and play a fundamental role in motor coordination and sensorimotor integration [8,43]. Experimental studies have shown that oxidative stress, excitotoxicity, and inflammatory signaling contribute to Purkinje cell degeneration following ischemic injury [8,43]. Consistent with these observations, the I/R group exhibited substantial Purkinje cell loss together with reduced Purkinje cell density, soma size, and layer thickness. Importantly, these structural alterations were paralleled by impaired performance in beam walking and grid walking tests. Because cerebellar function is critically dependent on the integrity of Purkinje cell circuitry, the behavioral deficits observed in the untreated ischemia group are likely to reflect underlying cerebellar dysfunction. The improvement in motor coordination and sensorimotor performance following skimmianine treatment therefore provides functional support for the histological evidence of neuroprotection. Rather than representing isolated histological findings, preservation of Purkinje cell architecture appears to translate into measurable functional benefits.

Beyond neuronal protection, the present study provides evidence that skimmianine may also preserve white matter integrity following ischemic injury. White matter damage and demyelination are increasingly recognized as important contributors to long-term neurological dysfunction after stroke [44]. Pro-inflammatory cytokines, oxidative stress, activated microglia, and oligodendrocyte injury collectively promote myelin degeneration and disruption of axonal conduction [42,45]. Transmission electron microscopy demonstrated severe myelin abnormalities in untreated ischemic animals, including vacuolization, lamellar separation, reduced myelin thickness, and increased g-ratio values. Quantitative ultrastructural analysis further confirmed significant deterioration of myelin integrity after I/R. In contrast, skimmianine treatment substantially improved myelin thickness, reduced vacuolar degeneration, and normalized g-ratio values. These findings suggest that suppression of oxidative and inflammatory injury may indirectly preserve oligodendrocyte function and myelin organization. Interestingly, systemic TNF inhibition has recently been reported to improve myelin integrity and neurological recovery after experimental stroke [42], supporting the possibility that the reduction in TNF-α signaling observed in the present study contributed to the preservation of cerebellar white matter.

The network pharmacology and molecular docking analyses provide additional mechanistic context for the experimental findings. The overlapping target network identified several inflammatory mediators, including TNF, IL6, IL1B, TLR4, STAT3, and IFNG, all of which are closely associated with neuroinflammatory signaling after ischemic injury [13,16]. Enrichment analyses further highlighted biological processes related to inflammatory responses, cellular stress, and immune activation. Although these computational findings cannot establish causality, they support the biological plausibility of the observed anti-inflammatory effects and suggest that skimmianine may act through multiple interconnected signaling pathways rather than a single molecular target. Similarly, molecular docking demonstrated a moderate predicted interaction between skimmianine and TNF-α. It should be noted that the enrichment of inflammation-related pathways was expected to some extent because the analysis was intentionally centered on TNF-α-associated proteins. Therefore, the primary value of the enrichment analysis was to identify the specific biological processes represented within the overlapping target network rather than to establish the involvement of inflammation itself. However, docking analysis alone cannot establish functional inhibition or direct modulation of TNF-α signaling. Therefore, these findings should be interpreted as preliminary computational evidence suggesting a potential molecular interaction that requires further experimental validation. Taken together, the experimental and computational findings indicate that skimmianine likely exerts pleiotropic effects involving simultaneous modulation of oxidative stress, neuroinflammation, neuronal survival, and myelin preservation.

Although cerebellar Iba1 and GFAP immunohistochemistry was not performed, several findings support the presence of a local anti-inflammatory effect within cerebellar tissue. Skimmianine treatment reduced cerebellar TNF-α immunoreactivity, preserved Purkinje cell morphology, improved motor coordination, and attenuated myelin ultrastructural damage [46,47]. Previous studies have demonstrated that inflammatory mediators, particularly TNF-α-associated signaling pathways, contribute to Purkinje cell degeneration, neuroinflammatory injury, and myelin disruption within the cerebellum and other regions of the central nervous system [42,45,46,47]. Therefore, the concomitant improvement in inflammatory, histological, behavioral, and ultrastructural parameters observed in the present study suggests that suppression of inflammatory injury within the cerebellum likely contributed to the neuroprotective effects of skimmianine. Nevertheless, future studies incorporating cerebellar Iba1 and GFAP immunohistochemical analyses will be necessary to directly characterize local glial responses.

Several limitations should be considered when interpreting the present findings. First, skimmianine was administered as a pretreatment regimen and evaluated at a single dose and a single reperfusion time point (23 h). Therefore, the present study primarily assesses prophylactic neuroprotection and does not provide information regarding the optimal therapeutic dose, treatment window, or long-term efficacy. Second, although MCAO is a well-established model of cerebral ischemia, the cerebellar alterations observed in this study reflect secondary remote pathological processes rather than direct cerebellar ischemia. Third, serum Iba1 and GFAP measurements reflect systemic neuroinflammatory and glial injury-related responses but do not provide direct evidence of cerebellar-specific microglial or astrocytic activation. Tissue-level validation using cerebellar Iba1 and GFAP immunohistochemistry or protein expression analyses would strengthen the interpretation of local glial responses. Fourth, behavioral assessments were performed during the acute reperfusion period and therefore reflect early sensorimotor impairment rather than long-term functional outcome. Finally, only female rats were included, and estrous cycle stages were not monitored, which may limit the generalizability of the findings. In addition, the network pharmacology and bioinformatic analyses should be regarded as hypothesis-generating and require further experimental validation. Future studies incorporating both sexes, longer follow-up periods, post-ischemic treatment paradigms, and detailed molecular investigations are warranted to further clarify the mechanisms underlying the neuroprotective effects of skimmianine.

Overall, the present findings suggest that skimmianine attenuates secondary cerebellar injury following cerebral I/R through coordinated modulation of oxidative stress, microglial activation, inflammatory signaling, neuronal preservation, and myelin integrity. These observations provide a rationale for future translational studies investigating skimmianine as a potential adjunctive neuroprotective strategy in ischemic stroke.

## 5. Conclusions

The present study demonstrates that skimmianine pretreatment mitigates secondary cerebellar injury following cerebral ischemia/reperfusion by attenuating oxidative stress, suppressing neuroinflammatory responses, and preserving both neuronal and white matter integrity. The reduction in TNF-α-, Iba1-, and GFAP-associated inflammatory alterations was accompanied by improved Purkinje cell preservation, enhanced motor performance, and maintenance of myelin ultrastructure. Network pharmacology and molecular docking analyses further supported the involvement of inflammation-related signaling pathways in the observed protective effects. Collectively, these findings suggest that skimmianine may represent a promising neuroprotective candidate for limiting remote cerebellar consequences of cerebral ischemia/reperfusion injury. Further studies using post-ischemic treatment paradigms and detailed molecular analyses are warranted to clarify its therapeutic potential and underlying mechanisms.

## Figures and Tables

**Table 1:** Behavioral assessment of cerebellar motor coordination and sensorimotor function in experimental groups.

Parameter	Sham	Skimmianine	I/R	I/R + Skimmianine	F-Value	*p*-Value
Beam Crossing Time (s)	4.8 ± 0.7	4.5 ± 0.7	11.7 ± 2.2 *	7.2 ± 1.3 #	39.52	<0.001
Beam Foot Slips (n)	0.6 ± 0.5	0.5 ± 0.5	5.9 ± 1.1 *	2.8 ± 0.7 #	84.48	<0.001
Grid Total Steps (n)	52.4 ± 5.0	54.1 ± 5.4	41.3 ± 5.1 *	47.8 ± 4.7 #	12.61	<0.001
Grid Foot Faults (n)	1.8 ± 0.7	1.7 ± 0.5	7.8 ± 1.7 *	4.1 ± 0.8 #	50.78	<0.001
Grid Foot Fault Rate (%)	3.4 ± 1.1	3.1 ± 1.0	18.6 ± 4.0 *	8.7 ± 2.5 #	58.44	<0.001

Data are presented as mean ± SD. One-way ANOVA followed by Tukey’s post hoc test. * *p* < 0.05 vs. Sham group; # *p* < 0.05 vs. I/R group.

**Table 2:** Effects of Skimmianine on serum TAS, TOS, and oxidative stress index levels in experimental groups.

Parameter	Sham	Skimmianine	I/R	I/R + Skimmianine	F-Value	*p*-Value
TAS (mmol Trolox Eq/L)	1.52 ± 0.18	1.56 ± 0.21	0.89 ± 0.15 *	1.29 ± 0.20 #	24.91	<0.001
TOS (µmol H_2_O_2_ Eq/L)	6.80 ± 1.41	6.40 ± 1.26	15.60 ± 3.03 *	9.20 ± 2.04 #	31.92	<0.001
OSI	4.43 ± 0.47	4.07 ± 0.31	17.48 ± 0.50 *	7.07 ± 0.47 #	1549.58	<0.001

Data are presented as mean ± SD. One-way ANOVA followed by Tukey’s post hoc test. * *p* < 0.05 vs. Sham group; # *p* < 0.05 vs. I/R group.

**Table 3:** Effects of Skimmianine on serum neuroinflammatory markers in experimental groups.

Parameter	Sham	Skimmianine	I/R	I/R + Skimmianine	F-Value	*p*-Value
TNF-α (pg/mL)	18.6 ± 4.4	20.1 ± 5.0	68.4 ± 10.2 *	34.9 ± 7.4 #	84.52	<0.001
Iba1 (pg/mL)	11.7 ± 1.6	14.5 ± 2.2	41.3 ± 9.7 *	24.2 ± 6.9 #	38.75	<0.001
GFAP (ng/mL)	87.7 ± 9.5	103.5 ± 18.1	334.5 ± 12.5 *	237.0 ± 7.9 #	686.83	<0.001

Data are presented as mean ± SD. Statistical comparisons were performed using one-way ANOVA followed by Tukey’s post hoc test. TNF-α: tumor necrosis factor-alpha; Iba1: ionized calcium-binding adaptor molecule 1; GFAP: glial fibrillary acidic protein. * *p* < 0.05 vs. Sham group; # *p* < 0.05 vs. I/R group.

**Table 4:** Semi-Quantitative Histopathological Scores and TNF-α Expression in Cerebellar Tissue.

Parameter	Sham	Skimmianine	I/R	I/R + Skimmianine	F-Value	*p*-Value
Purkinje cell loss	0.00 ± 0.00	0.13 ± 0.35	2.62 ± 0.52 *	1.12 ± 0.48 #	98.87	<0.001
Edema	0.12 ± 0.35	0.25 ± 0.46	2.75 ± 0.46 *	1.25 ± 0.71 #	58.71	<0.001
Vascular congestion	0.00 ± 0.00	0.12 ± 0.35	2.37 ± 0.52 *	1.00 ± 0.76 #	54.17	<0.001
TNF-α expression	0.25 ± 0.46	0.37 ± 0.52	2.62 ± 0.74 *	1.12 ± 0.83 #	26.70	<0.001

Data are presented as mean ± SD. Overall group comparisons were performed using one-way ANOVA followed by Tukey’s post hoc test. * *p* < 0.05 vs. Sham group; # *p* < 0.05 vs. I/R group.

**Table 5:** Quantitative analysis of Purkinje cell density, soma area, and Purkinje layer thickness in experimental groups.

Parameter	Sham	Skimmianine	I/R	I/R + Skimmianine	F-Value	*p*-Value
Purkinje Cell Count (/mm)	18.4 ± 1.5	18.9 ± 1.2	9.8 ± 1.7 *	14.7 ± 1.5 #	64.07	<0.001
Purkinje Cell Soma Area (µm^2^)	312.6 ± 22.7	318.2 ± 18.8	201.4 ± 18.0 *	258.9 ± 18.6 #	61.88	<0.001
Purkinje Layer Thickness (µm)	28.5 ± 2.3	29.1 ± 1.9	17.2 ± 2.7 *	23.6 ± 2.4 #	43.66	<0.001

Data are presented as mean ± SD. One-way ANOVA followed by Tukey’s post hoc test. * *p* < 0.05 vs. Sham group; # *p* < 0.05 vs. I/R group.

**Table 6:** Quantitative ultrastructural analysis of myelinated fibers in experimental groups.

Parameter	Sham	Skimmianine	I/R	I/R + Skimmianine	F-Value	*p*-Value
Axon Diameter (µm)	1.84 ± 0.18	1.88 ± 0.17	1.79 ± 0.21	1.82 ± 0.18	0.39	0.760
Fiber Diameter (µm)	2.91 ± 0.24	2.97 ± 0.29	2.38 ± 0.24 *	2.67 ± 0.27 #	8.31	<0.001
Myelin Thickness (µm)	0.53 ± 0.07	0.54 ± 0.06	0.29 ± 0.05 *	0.42 ± 0.07 #	34.72	<0.001
G-ratio	0.63 ± 0.04	0.63 ± 0.03	0.75 ± 0.05 *	0.68 ± 0.05 #	14.85	<0.001
Vacuolized Fibers (%)	3.20 ± 1.10	3.00 ± 1.10	24.70 ± 4.20 *	11.20 ± 2.80 #	123.51	<0.001

Data are presented as mean ± SD. Overall group comparisons were performed using one-way ANOVA followed by Tukey’s post hoc test. * *p* < 0.05 vs. Sham group; # *p* < 0.05 vs. I/R group.

## Data Availability

The data presented in this study are available on request from the corresponding author and publicly not available due to ethical restrictions.
